# Out-of-pocket medical expenses compared across five years for patients with one of five common cancers in Australia

**DOI:** 10.1186/s12885-021-08756-x

**Published:** 2021-09-25

**Authors:** Astrid J. Rodriguez-Acevedo, Raymond J. Chan, Catherine M. Olsen, Nirmala Pandeya, David C. Whiteman, Louisa G. Gordon

**Affiliations:** 1 QIMR Berghofer Medical Research Institute, Population Health Department, Herston, Brisbane, Q4006 Australia; 2 Queensland University of Technology (QUT), School of Nursing and Cancer and Palliative Care Outcomes Centre, Kelvin Grove, Brisbane, Q4059 Australia; 3 Princess Alexandra Hospital, Division of Cancer Services, Wollonggabba , Brisbane, Q4102 Australia; 4 The University of Queensland, Faculty of Medicine, Herston, Brisbane, Q4006 Australia

**Keywords:** cancer, Out-of-pocket medical costs, Population-based, Private health insurance

## Abstract

**Background:**

Patient medical out-of-pocket expenses are thought to be rising worldwide yet data describing trends over time is scant. We evaluated trends of out-of-pocket expenses for patients in Australia with one of five major cancers in the first-year after diagnosis.

**Methods:**

Participants from the QSKIN Sun and Health prospective cohort Study with a histologically confirmed breast, colorectal, lung, melanoma, or prostate cancer diagnosed between 2011 and 2015 were included (*n* = 1965). Medicare claims data on out-of-pocket expenses were analysed using a two-part model adjusted for year of diagnosis, health insurance status, age and education level. Fisher price and quantity indexes were also calculated to assess prices and volumes separately.

**Results:**

On average, patients with cancer diagnosed in 2015 spent 70% more out-of-pocket on direct medical expenses than those diagnosed in 2011. Out-of-pocket expenses increased significantly for patients with breast cancer (mean AU$2513 in 2011 to AU$6802 in 2015). Out-of-pocket expenses were higher overall for individuals with private health insurance. For prostate cancer, expenses increased for those without private health insurance over time (mean AU$1586 in 2011 to AU$4748 in 2014) and remained stable for those with private health insurance (AU$4397 in 2011 to AU$5623 in 2015). There were progressive increases in prices and quantities of medical services for patients with melanoma, breast and lung cancer. For all cancers, prices increased for medicines and doctor attendances but fluctuated for other medical services.

**Conclusion:**

Out-of-pocket expenses for patients with cancer have increased substantially over time. Such increases were more pronounced for women with breast cancer and those without private health insurance. Increased out-of-pocket expenses arose from both higher prices and higher volumes of health services but differ by cancer type. Further efforts to monitor patient out-of-pocket costs and prevent health inequities are required.

**Supplementary Information:**

The online version contains supplementary material available at 10.1186/s12885-021-08756-x.

## Background

The societal cost of cancer is rising rapidly in many countries. In Australia, more than 145,000 people are expected to be diagnosed with cancer in the year 2020 (excluding non-melanoma or keratinocyte skin cancers) [1] and over one million people are currently living with cancer. Cancer exerts a considerable burden on individuals and the economy, which will continue to grow with greater service use, high-cost therapeutics and imaging, new technologies and, to a lesser extent, population aging [2, 3]. While many reports have documented the explosive costs of new technologies and therapies in cancer care [4], parallel evidence highlights the financial burden to patients and families and their reduced affordability to access healthcare services [5]. In many countries, the very high cost of targeted cancer therapies and immunotherapies causes great concern for families unable to afford them [6, 7].

Medical out-of-pocket expenses can be categorised into direct expenses, which include patient co-payments toward consultations, tests, procedures and medications, and indirect or non-medical expenses, including transport, parking, and accommodation costs necessary to receive healthcare. While the direct medical out-of-pocket expenses have commonly been estimated in the immediate period following a cancer diagnosis, the indirect costs are also substantial and can quickly accummulate. Overall, patients with cancer use a high number of healthcare services and medicines and have higher medical out-of-pocket expenses compared with patients without cancer, irrespective of whether they have private health insurance or not [6, 7]. The highest proportion of medical out-of-pocket expenses has been reported for surgery and investigations [6, 8, 9], while the greatest indirect expenses are travel-related for patients living in rural or remote areas [9–11].

Financial hardship has been shown to affect patients‘access and adherence to treatment with some patients missing appointments, delaying, or foregoing treatment, which may lead to poorer health outcomes [12–15]. The stress caused by high out-of-pocket expenses coupled with lost employment and reduced income can exacerbate cancer symptoms [16], adversely affect patients’ quality of life and that of their immediate family or informal carers [17, 18]. The known risk factors of high financial burden include: younger age, being female, adjuvant therapies, advanced disease, low income, and living away from treatment centres [5]. Furthermore, awareness of forced retirement [19] or inability to stop work due to financial need [20] are problems arising more commonly in cancer populations.

Australia has a mixed public-private health system whereby medical services provided outside of public hospitals are delivered privately. Medicare is Australia’s universal health insurance scheme available to all Australian citizens and permanent residents. It covers medical services listed in the Medical Benefits Schedule (MBS) and prescription medicines listed in the Pharmaceutical Benefits Scheme (PBS). Medicare does not cover services in public hospitals which are funded through individual State governments. Doctors and other health professionals operating in private practice may charge what they believe is fair and reasonable [21]. Each service is made up of the provider charge, the Medicare rebate (what the government pays) and the remainder is the patient’s out-of-pocket expense. Health professionals can choose not to charge above the Medicare rebate so that the patient does not incur a co-payment (termed ‘bulk-billing’). Advertising their services as ‘bulk billing’ is attractive for patients who wish to avoid out-of-pocket costs and may give health professionals a competitive advantage. Even if a patient is treated in a public hospital, patients with cancer in Australia access private providers (e.g., general practitioners, pathology services, pharmacists) and will incur out-of-pocket expenses unless the co-payments are fully covered. The latest Medicare reports indicate that bulk-billing rates differ among providers and are not available in all areas or for all patient groups [22]. Specialist visits have rates of bulk billing of ~ 30% [21]. In general, low-income individuals are more often bulk-billed [21] but less likely to see specialists [23] and co-payments represent a higher proportion of their disposable income. Further complicating the understanding of out-of-pocket expenses over time is the ‘Medicare freeze’ where the government ceased indexing Medicare rebates to inflation between 2014 and 2017, thereby increasing pressure on providers with rising operational costs to shift these costs onto patients.

Despite patients with cancer having high medical costs, there is very little research on whether out-of-pocket medical expenses have changed over time. There are also few routinely reported statistics on out-of-pocket expenses to inform policy [24]. Thus, we estimated the differences in out-of-pocket medical expenses across five years and associated drivers among individuals newly diagnosed with one of five dominant cancers.

## Methods

### Study design and participants

In 2010–2011, the QSKIN study recruited 43,794 residents of Queensland, Australia, aged 40–69 years selected at random from the Queensland Electoral Roll, 92% of whom (*n* = 40,438) gave consent to provide linked Medicare data [25]. Study participants representing the general population comprised 46% males (mean age of 57 years) and 54% females (mean age of 55 years), mostly of white European ancestry (93%). Participants from the QSKIN Sun and Health Study [25] with a histologically confirmed diagnosis of breast, colorectal, lung, melanoma, or prostate cancer between 2011 and 2015 were included in the present analysis. This analysis used individual-level linked data from the QSKIN survey [25], Queensland Cancer Registry records and Medicare claims records between 2011 and 2015. Self-completed baseline survey items used were sex, age, marital status, private health insurance, education, self-rated health, and body mass index. Only cases with at least one year of Medicare data were included in this analysis (*n* = 1965) and no patients had died within the first year. We excluded individuals with two or more cancer types, different from melanoma, diagnosed within one year and those with missing health insurance status or level of education (*n* = 196).

### Data sources

We used linked data from the baseline survey and Medicare MBS and PBS items processed for each participant between their date of consent and 30 June 2016. MBS and PBS data used in the analyses included the item number, provider fee, Medicare rebate (benefit paid), patient out-of-pocket cost, and date of service. The data incorporated all consultations, tests, imaging, procedures and pharmaceuticals billed through Medicare. All MBS and PBS items for each patient, rather than cancer-specific items, were used for this analysis because it is not possible to attribute generically worded items specifically to cancer. Out-of-pocket amounts are recorded for each service or medicine recorded by Medicare. However, for therapeutic services conducted in a private setting (including private hospitals), the amounts are prior to reimbursement by private health insurers. For the majority of the items, insurers do not reimburse out-of-hospital services such as doctors’ visits, medications, community-based imaging, pathology, or any out-of-pocket hospital services covered by Medicare. All costs were inflated to 2016 prices. The study was approved by the QIMR Berghofer Human Ethics Research Committee and all participants provided informed written consent to take part.

### Statistical analyses

Descriptive analyses were used to present the cancer type by year of diagnosis and their baseline socio-demographic characteristics using frequencies and percentages for categorical data and means and standard deviations for continuous variables. Chi-square tests and Fisher’s tests were performed to identify statistically significant differences in the baseline variables across cancer groups.

We calculated the total out-of-pocket expenses for all patients during the first year after diagnosis, and the mean cost per patient per year of diagnosis to evaluate annual fluctuations between 2011 and 2015 for those patients with out-of-pocket expenses. Patient out-of-pocket expenses were presented using medians and interquartile ranges (IQR) as well as means and standard deviations. To measure differences in out-of-pocket expenses each year, we performed a two-part model analysis [26] to account for the excess zeros from individuals with no out-of-pocket expenses during the first year of cancer treatment (i.e., they were fully bulk-billed for every service). In the two-part model, the first part includes a logistic regression fitted to the probability of observing a positive-versus-zero out-of-pocket cost, while the second part is conditional on a positive out-of-pocket cost, fits a generalized linear model (GLM) with a gamma family and log link. Diagnostic tests confirmed the appropriateness of the family and link parameters (Additional File 1). Variables from a full model (year of diagnosis, age group, sex, marital status, level of education, private health insurance status (yes/no), body mass index, drinks per week and self-health assessment (poor, good and excellent)) were excluded based on their statistical significance and the Akaike Information Criterion (AIC). The predicted values from the two-part models are presented and kernel density plots were constructed showing the adjusted distribution of the mean out-of-pocket expenses in 2011 and 2015 for all cancers. All analyses were performed using R. Statistical significance was considered at *p* < 0.05.

### Fisher Price and Quantity Index

Calculating trends in out-of-pocket expenses can be attributed to rising prices and/or higher quantities of services or products (existing or new). To evaluate whether prices were rising for each category of service, the year-to-year average change from the base year was calculated using the Fisher Price Index, a measure of the average price level based on a basket of goods that allow us to estimate movements in the price over time [27, 28]. Similarly, a Fisher Quantity Index calculates the average quantities of Medicare items used per patient over time [27]. The formulas used to derived these indices are presented in-depth by Hua, et al. [27] and presented in Additional File 2. These indexes clarify if out-of-pocket changes are due to more expensive items or the frequency of services. The calculation of the price and quantity indexes required MBS items codes claimed from 2011 to through 2015. Therefore, new items absent in 2011, and old items absent or replaced in 2015, were excluded from this analysis.

## Results

### Total first-year costs

A total of 1965 participants comprising: 852 (44%) patients with melanoma, 451 (23%) with prostate cancer, 396 (19%) with breast cancer, 160 (8%) with colorectal cancer and 106 (5%) with lung cancer (Table 1), were included. Overall, 55% were male, the mean age was 59.6 (SD 9.4) (lowest for breast cancer (57.4) and highest for prostate (61.7)), 70% had private health insurance and 55% had a high education level (Tertiary degree or higher). The overall adjusted mean out-of-pocket expenses during the first year from diagnosis for all patients with cancer between 2011 and 2015 was AU$2489 (SD $1932; IQR AU$1114–$4056) (Additional File 3). A small proportion of patients (*n* = 63, 3.2%) had zero out-of-pocket expenses for MBS services. Those diagnosed between 2013 and 2015, or who had private health insurance or a diagnosis of breast cancer, were up to six times more likely to have paid out-of-pocket expenses than patients diagnosed in 2011–2012, without private health insurance and with a diagnosis other than breast cancer. Adjusting for private health insurance status, year of diagnosis, age and level of education, mean out-of-pocket expenses for the first 12 months after diagnosis were highest for patients with prostate cancer (mean AU$4269; IQR AU$3012–$5639), colorectal cancer (mean AU$2725; IQR AU$466–$3365) and lung cancer (mean AU$2442; IQR AU$601–$3374) (Additional File 3).

**Table 1:** Sample Descriptives. Only variables with a significant effect on the out-of-pocket expenses in the analysed sample are presented

Cancer type	All cancers	Breast	Colorectal	Lung	Melanoma	Prostate
**Sample size (n)**	1965	396	160	106	852	451
**Age at a diagnosis (mean (SD))**	59.6 (9.41)	57.4 (7.82)	59.6 (7.28)	62.2 (6.91)	58.4 (7.99)	61.7 (5.71)
**Sex**						
Female	45%	99%	44%	47%	44%	0
Male	55%	1%	56%	53%	56%	100%
**Private Health Insurance**						
Yes	70%	70%	59%	47%	75%	72%
No	29%	30%	41%	53%	25%	27%
**Education**						
High school or lower	25%	25%	36%	47%	23%	22%
Technical, trade certificate	19%	26%	20%	17%	18%	16%
Tertiary degree or higher	55%	49%	43%	36%	58%	62%

### Cost trends over time

**Fig. 1:**
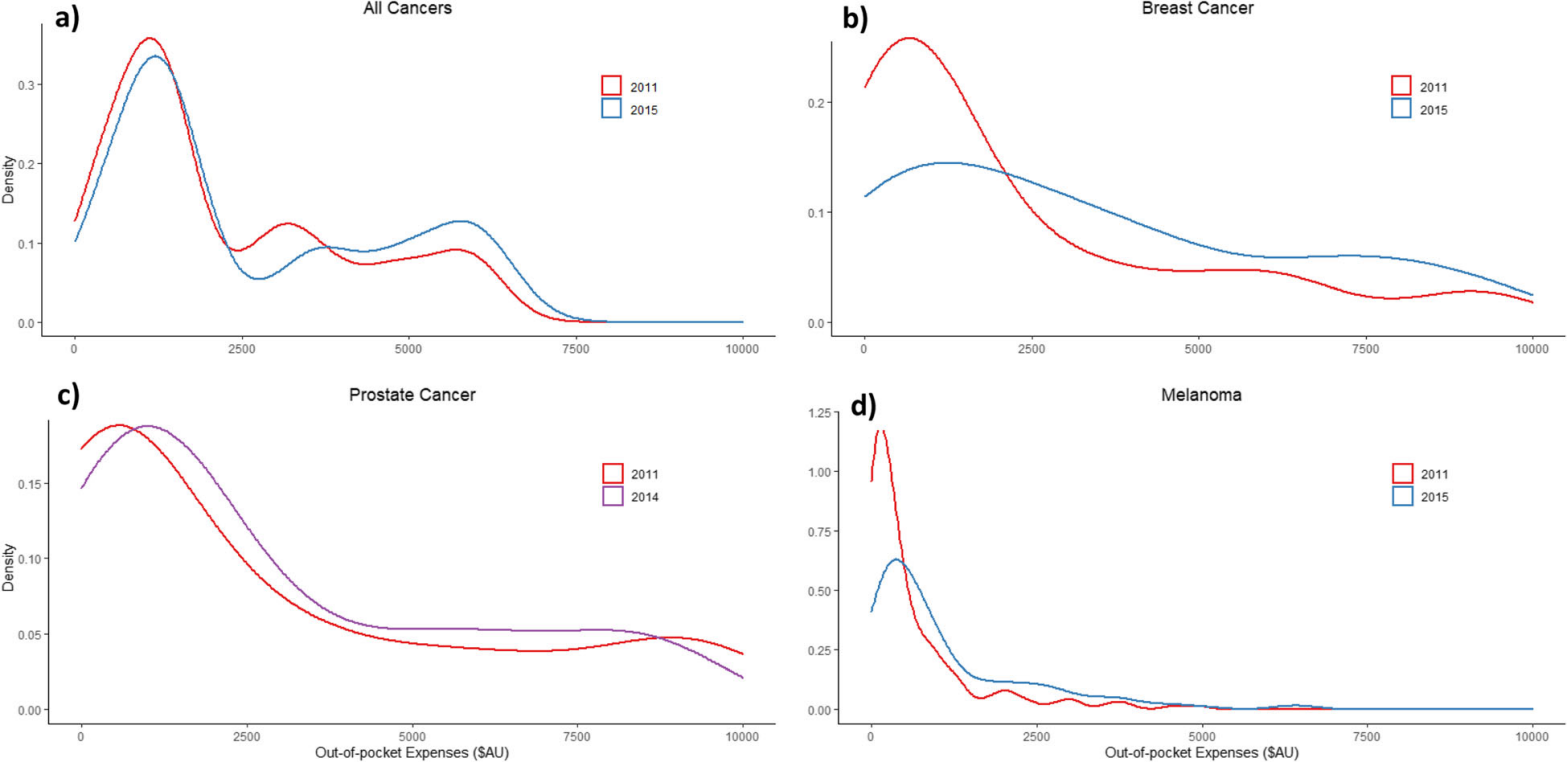
Density plots for the distribution of the predicted mean out-of-pocket expenses in 2011 and 2015. Density plot shows the underlying probability distribution of the two-part model predicted mean out-of-pocket expenses in 2011 and 2015 for all cancers, breast and prostate cancer and melanoma, using a kernel density estimation. The height of the curve is scaled such that the area under each curve equals one; the y-axis depends on the maximum out-of-pocket expenses values in the original input file (different for all plots, hence inconsistency in y-axis); Values in the y axis do not have any interpretative value, other than serving as a reference to compare the height of different curves in the same plot. . For all cancers, in 2011 and 2015 most of the out-pocket spending was less than AU$2500; however, the distribution peak is lower in 2015 compared to 2011, indicating fewer patients with expenses less than AU$2500. On the contrary, patients incurred higher expenses ($AU 2500 - $7000) more frequently during 2015 than 2011 (larger area under the curve in 2015 than 2011). For breast cancer, the 2015 curve is less pronounced indicating a somewhat even distribution of costs among most patients ranging from 0 to $AU7,500. Out-of-pocket expenses in prostate cancer, were higher in 2014, with no significant difference to 2015, compared to 2011. 2011 and 2014 curves have the same height but, in the latest year, the curve has moved towards the right, indicating higher prices during that year. Costs ranging from $AU 2500 to $AU8,000 were more common in 2014 compared to 2011. For melanoma, a peak around $AU 500 in 2011 expenses, twice as high as the peak in the 2015 distribution, shows how fewer individuals paid lower out-of-pocket expenses in 2015 compared to 2011. The density of 2011 costs, rapidly flattened from AU$ 2500 to zero, while those in 2015, showed a slow reduction until they flattened in $AU 5000 indicating higher proportion of individuals paying higher prices in 2015. The asymmetry in all plots reflects the typically skewed distribution of cost data, in this case, the fitted gamma distribution. Distributions are adjusted by: a) All cancers: health insurance, sex, age, level of education, cancer type and Year b) Breast cancer: Health insurance, age, year c) Prostate cancer: Health insurance and year d) Melanoma: health insurance, year, level of education

On average, patients with cancer diagnosed in 2015 spent 70% more out-of-pocket on medical services and pharmaceuticals than those with a diagnosis in 2011 (Additional File 4). For all cancers, in 2011 and 2015 most of the out-pocket spending was less than AU$2500 (Fig. 1a);. Conversely, patients incurred higher expenses ($AU 3500 - $AU 7000) more frequently during 2015 than 2011 (Fig. 1a). Out-of-pocket expenses significantly increased over the years 2011–2015 for patients with breast cancer and melanoma (Fig. 1, Table 2). The mean adjusted spending for patients with melanoma in 2015 was AU$1043 (Additional File 3) with individuals paying an average of AU$620 more than in 2011 (Table 2). Among individuals with out-of-pocket expenses, those with breast cancer diagnosed in 2015 paid AU$4289 more than patients diagnosed in 2011 (AU$2513) (Table 2). Out-of-pocket expenses for men with prostate cancer, were similar in 2014 and 2015, and both higher compared with 2011. Fig. 1c curves in 2011 and 2014 are the same height but in 2014 moved towards the right, indicating higher out-of-pocket costs during that year ranging from $AU2,500 to $AU8,000. A significant interaction between year and private health insurance status was found when we modelled out-of-pocket expenses for patients with prostate cancer. Therefore, we modelled out-of-pocket cost independently for health insurance status (yes and no) to understand the effect of health insurance over time. For prostate cancer, there was a significant increase in out-of-pocket expenses over time for individuals without private health insurance, who paid an average of AU$4748 in 2014, 3-fold higher than for those diagnosed in 2011 (AU$1586) (data not shown). Out-of-pocket expenses were stable over time for health insurance holders. Patients with colorectal cancer spent more in 2012 and 2014 compared to 2011 but expenses were similar in 2011 and 2015 (Table 2).

**Table 2:** Generalized Linear Model results for all non-zero total out-of-pocket expenses ($AUD) after one year of a major cancer diagnosis. The second part of the two-part model is presented only (3.2% of patients had $0 out-of-pocket expenses) and the first part is presented in the Additional File 5. Coeff: Regression coefficient; Ratio:exp. (Coeff)-1 this is the cost ratio of the response level compared to the referent in the variable; Extra cost: Extra cost paid with respect to the mean of the reference group (ref) in each categorical variable; Statistical Significance; * < 0.05; ** < 0.001; *** < 0.0001

	Breast			Colorectal			Lung			Prostate			Melanoma		
Variables	Cost Ratio	95% CI	Extra Cost ($AU)	Cost Ratio	95% CI	Extra Cost ($AU)	Cost Ratio	95% CI	Extra Cost ($AU)	Cost Ratio	95% CI	Extra cost ($AU)	Cost Ratio	95% CI	Extra Cost ($AU)
**Health Insurance**															
No – ref. (mean (sd))	ref	–	867.36 (1359)	ref	–	536 (698)	ref	–	621 (827)	ref	–	1586 (2546)	ref	–	416 (482)
Yes	3.01	1.86, 4.63	2606.5***	8.05	3.50, 16.3	4315.94***	9.75	2.34, 28.01	6054.31***	6.33	3.21, 11.05	10,038.97***	1.91	1.40, 2.50	793.60***
**Year**															
2011 (ref)	ref	–	2513 (2948)	ref	–	2344 (2675)	ref	–	2413 (1813)	ref	–	3692 (3981)	ref	–	657 (895)
2012	0.89	0.35, 1.66	2239.34*	22.26	3.00, 116.61	52175***	−0.07	−0.72, 1.89	−160.41	2.36	0.84, 4.91	8697.61***	0.85	0.47, 1.34	561.02***
2013	0.84	0.31, 1.58	2099.35*	3.50	−0.15, 17.11	8195.80	0.12	−0.63, 1.85	297.33	3.15	1.08, 7.34	11,620.79***	0.71	0.36, 1.16	469.44***
2014	1.29	0.63, 2.22	3244.93**	11.12	1.23, 50.67	26,066.50**	3.11	0.31, 9.89	7516.11**	2.01	0.58, 4.63	7426.59***	0.75	0.37, 1.23	489.85***
2015	1.71	0.93, 2.80	4289.34***	0.32	−0.42,1.97	741.33	1.16	−0.31, 4.71	2794.19	0.38	−0.28, 1.61	1418.97	0.94	0.52, 1.48	620.21***
**Age**															
< 50 (ref)	ref	–	4571.00	ref	–	2787 (3230)									
50–60	0.36	−0.03, 0.91	1639.81	5.28	0.32, 21.18	14,722.89**									
> 60	0.28	−0.09, 0.79	1261.71	4.26	−0.52, 1.80	11,879.50**									
**Education**															
Tertiary degree or higher													ref	–	731 (1022)
Technical, trade certificate													−0.07	−0.24, 0.14	−50.03
High school or lower													−0.19	−0.33, − 0.02	− 139.69
**Health Insurance**: **Year**															
No:2011 (ref)	ref		518 (442)	ref	–	390 (262)	ref	–	303 (126)	ref		599 (831)			
Yes:2012	−0.04	−0.32, 0.35	−19.81	0.71	−0.33, 3.43	275.77	0.07	−0.73, 3.43	42.66*	−0.61	−0.80, − 0.24	− 367.96**			
Yes:2013	− 0.08	− 0.34, 0.30	−39.63	0.16	− 0.52, 1.80	60.54	0.45	− 0.58, 4.71	280.69	− 0.67	− 0.85, − 0.28	−400.79**			
Yes:2014	− 0.29	−0.49, 0.00	− 149.09	− 0.21	−0.68, 0.96	−82.35	− 0.75	−0.93, − 0.03	− 463.18	−0.66	− 0.84, − 0.30	− 396.60**			
Yes:2015	− 0.33	−0.52, − 0.06	− 171.59	−0.26	− 0.71, 0.99	−100.13	−0.21	− 0.78, 2.09	− 130.62	−0.08	− 0.55, 0.91	−45.72			
**Health Insurance: Age**															
No: < 50 (ref)	ref		3118 (3159)												
Yes: 50–60	−0.19	−0.42, 0.14	− 579.99												
Yes: > 60	−0.03	−0.31, 0.37	−78.90												
**Year: Age**															
2011: < 50 (ref)				ref	–	641 (829)									
2012: 50–60				−0.97	−0.99, − 0.79	− 621.05***									
2013: 50–60				− 0.86	− 0.97, − 0.21	− 549.10*									
2014: 50–60				− 0.91	− 0.98, − 0.50	− 584.07**									
2015: 50–60				1.03	− 0.31, 4.74	657.84									
2012: > 60				− 0.96	−0.99, − 0.78	− 616.25***									
2013: > 60				−0.82	− 0.95, − 0.09	−522.43*									
2014: > 60				− 0.88	−0.97, − 0.38	− 564.70**									
2015: > 60															

### Factors predicting higher out-of-pocket expenses

The year that expenses were incurred influenced whether patients experienced zero or positive out-of-pocket expenses, with latter years having a lower (but non-significant) proportion of patients with zero expenses than in earlier years (Additional File 5). Across all cancers, having private health insurance was a significant predictor of higher out-of-pocket expenses, up to nine times higher in lung cancer (Table 2) compared with patients without private health insurance. Furthermore, older individuals (50+ years old) with colorectal cancer incurred higher out-of-pocket expenses than younger patients (40–50 year old) (< 50 yo: AU$2787; 50–60 yo: AU$17,509; > 60 yo: AU$14,666). . Age did not significantly predict the out-of-pocket expenses in melanoma, lung, and prostate cancer patients, although QSKIN participants were all aged between 40 and 70 years at recruitment in 2011. Lower levels of education were predictors of lower costs for prostate cancer (Tertiary degree: AU$731; Technical Education: AU$681; High School: AU$592)). Across all years, health insurance holders spent 60% of the total out-of-pocket cost on therapeutic procedures and 14% on pharmaceuticals while in contrast, patients with cancer without health insurance spent an average of 40% on pharmaceuticals and 38% on therapeutic procedures (Fig. 3). Similar proportions of cost category expenses occurred by different cancer types (not shown).

### Prices (out-of-pocket cost values) versus quantities

**Fig. 2:**
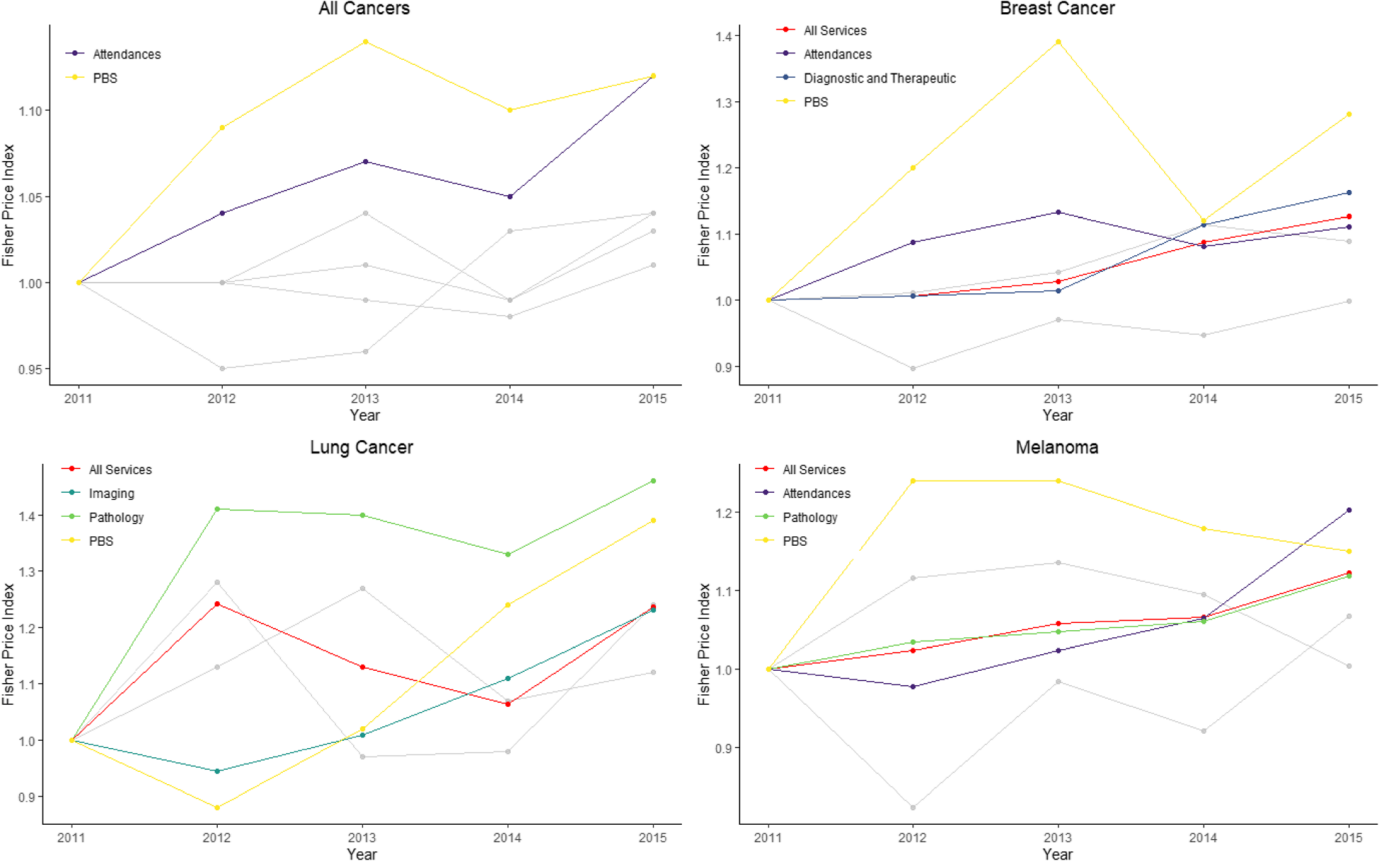
Fisher Price Index. To evaluate whether prices were rising for each category of service, the year-to-year average change from the base year was calculated using the Fisher Price Index. Coloured lines indicate health service categories (All Services, Attendances, Diagnostic and Therapeutic, Diagnostic Imaging, Pathology and all PBS) whose price increased over time. Grey lines indicated health services whose price remained the same or decreased over time

For all cancers, prices increased over the years for PBS medicines and doctor attendances (Fig. 2) but fluctuated for other MBS services. For patients with breast cancer, there was a progressive increase from 2011 to 2014 in prices for services (Fig. 2, Additional File 6–7); and a sharp increase in number of services until 2014, with a drop in 2015. Price increases were more pronounced for diagnostic services and therapeutic procedures (e.g. surgery, radiation therapies). Patients with colorectal cancer experienced higher index prices during 2012–2013 while quantity indexes slowly dropped from 2012 through to 2015. Out-of-pocket costs paid for pathology and all MBS services increased for lung cancer patients and melanoma patients between 2011 and 2015. Melanoma patients had fewer MBS services over time between 2012 and 2015. This, however, might reflect the introduction of new items in 2015, replacing the services provided in 2011, rather than an actual decrease in the use of services over time. PBS prices increased for patients with breast and lung cancer, melanoma, and for all cancers combined.

**Fig. 3:**
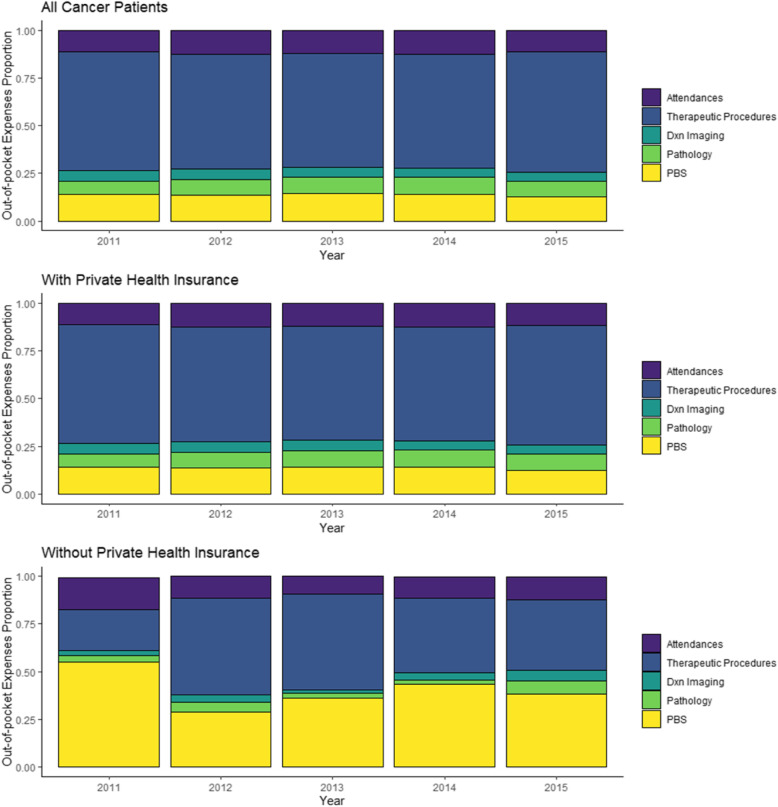
MBS and PBS out of pocket spending. Distribution of out-of-pocket cost by MBS subcategories and PBS spending for all cancer patients together (top panel), those with (middle panel) and without (bottom panel) health insurance

## Discussion

We evaluated all out-of-pocket medical expenses in the first-year after diagnosis for Australian patients with cancer over a five-year period. On average, patients with a cancer diagnosis between 2011 and 2015, paid AU$2462 in out-of-pocket expenses. The highest and lowest medical expenses paid were for individuals diagnosed with prostate cancer and melanoma, respectively. Over 2011–2015, out-of-pocket expenses for all medical services almost tripled for breast cancer patients and doubled for melanoma patients. Changing prices of diagnostic and therapeutic procedures, medical attendances, and diagnostic imaging, as well as increased frequency in pathology investigations and PBS items, were the main drivers of higher expenses.

A detailed report on 1919 Australian patients with breast cancer, surveyed in 2016, found mean 5-year costs of $4809 with an interquartile range from $1510 to $17,200 [29]. Some of the high-cost items borne by patients represent new technologies that are not billable items on the MBS (and excluded from this study) including Oncotype Dx and other genetic tests, MRIs and radiotherapy. Like our report, studies show private health insurance is associated with higher out-of-pocket costs. Saxby et al. (2020) reported lower out-of-pocket costs for diagnosis (by mean AU$741, 95%CI $316–$1180) for services covered for free within the national BreastScreen service than for community-detected breast cancers by private health insurance holders [30]. Out-of-pocket expenses for lung cancer patients tripled from 2011 to 2014, with the 2014 adjusted mean cost of AU$2624. This is a higher estimate than AU$1721 (€890, calculated in 2014, AU$1 = €0.49) [31] previously reported for stage IV non-small cell lung cancer in Europe [32]. However, caution should be taken when interpreting lung cancer estimates because of the small sample size (*n* = 100) in our analysis. Colorectal cancer mean estimates (AU$2725, IQR = AU$466–$3365) were comparable with €1589 (AU$3242) reported in Europe [33].

Our study showed that out-of-pocket costs were substantially higher in patients with private health insurance but they were stable over time, whereas out-of-pocket costs increased for those without private health insurance. We also show that patients without private health insurance, usually made up of more individuals from lower socio-economic backgrounds, have a higher proportion of their healthcare expenditure going towards medicines which are already heavily subsidised for Australians. Rana et al. [23] reported that those without private health insurance access fewer specialist visits but more general practitioner services. It is important to continue monitoring these differences in out-of-pocket expenses over time, and by subgroups, to understand whether systemic variations in healthcare exist in Australia.

There are some limitations of our study. Our data is now five years old and changes to Medicare items and PBS medications introduced since 2015 were not captured. Also, the introduction or exclusion of items during 2011–2015 could not be included in the price and quantity index analyses and additional items will have influenced cost differences between 2011 and 2015. We contend this will be small contributor to overall costs since, firstly, these apply to patients with metastatic cancer only and secondly, PBS medicines only attract a small co-payment. Overall, surgical procedures are the key cost drivers of overall out-of-pockets and these have not changed during this period. The out-of-pocket expenses listed in our data do not include reimbursements made by private health insurance companies although these would only apply to therapeutic procedures in private hospitals. In addition, indirect medical expenses were not reported here such as the additional costs such as travel, parking, paid home help, childcare services and income lost through needing time off work. These additional costs can mount quickly and are distressing especially for individuals living in rural areas needing to travel long distances to treatment centers. In this sense, our overall patient expenses reported here are conservative. The analyses could have used a GLM model after removing the small proportion of patients with zero costs in the Medicare dataset. However, since there is no stated percentage of zeros when an analyst should use a two-part model [34], we chose to include all patients with zero and positive expenses in a two-part model.

Although the distribution of the cancer types in this study do not represent the relative incidence of these five cancers according to Queensland cancer registrations, our objective was to assess changes over time by cancer type rather than observe incidence-based costs of our cancer sample. Furthermore, we cannot rule out potential differences in provider fees charged in other metropolitan and regional settings around Australia, which impact on the out-of-pocket findings in our study. These provider fees have been found to be higher in New South Wales and Victoria [29]. We acknowledge that not all possible drivers of rising out-of-pocket costs are captured in this study. One possible explanation is increased operational costs of private providers and stable Medicare rebates during (2011–17) which might explain higher out-of-pocket costs. Also costs may differ according to stage of cancer but we were unable to assess this in our study. Finally, Medicare administrative data used for this analysis were not able to be verified by secondary sources and although we have no reason to suspect anomalies, the data are not immune to errors [35].

It is important to routinely monitor out-of-pocket medical expenses as an essential part of assessing health system performance, even in countries with universal health care [36]. One study reported catastrophic spending (defined as spending more than 10% of household income on medical care) rose from 7 to 13% in low-income Australian households between 2006 and 2014 [36]. These outcomes substantially increase the risk of patients forgoing care due to affordability issues and increase health inequalities [37].

## Conclusion

Among patients diagnosed with one of the five prevalent cancers in Australia, overall first-year out-of-pocket medical expenses increased 70% from 2011 to 2015. Patients with breast cancer and melanoma experienced the largest increases in out-of-pocket spending while prostate cancer and colorectal cancer had high overall mean expenses during 2011–2015. Those without private health insurance faced expenses progressively and significantly higher over time and may be at risk of reduced access to healthcare.

## Supplementary Information

**Additional file 1.** Density plots for the tested gamma, lognormal and normal distributions.

**Additional file 2.** Calculation of the Fisher indices.

**Additional file 3.** Adjusted and unadjusted mean out-of-pocket expenses per year per cancer type.

**Additional file 4.** Generalized Liner Model (GLM) results for all cancers.

**Additional file 5.** First part of two-part model by cancer type.

**Additional file 6.** Price and Quantity Index.

## Data Availability

The data that support the findings of this study are available from QSkin study but restrictions apply to the availability of these data, which were used under license for the current study, and so are not publicly available.

## Abbreviations

AIC: Akaike Information Criterion
AU: Australian
GLM: Generalized linear model
IQR: Interquartile range
MBS: Medical Benefits Schedule
MRI: Magnetic resonance imaging
QIMR: Queensland Institute of Medical Research
PBS: Pharmaceutical Benefits Scheme
