# Supplementary material for: Out-of-pocket medical expenses compared across five years for patients with one of five common cancers in Australia
Source: BMC Cancer. 2021 Sep 25;21:1055. doi: 10.1186/s12885-021-08756-x (PMC8466922; doi:10.1186/s12885-021-08756-x)
Supplement: Supplementary file 1 — Additional file 1. Density plots for the tested gamma, lognormal and normal distributions. [file 12885_2021_8756_MOESM1_ESM.docx]

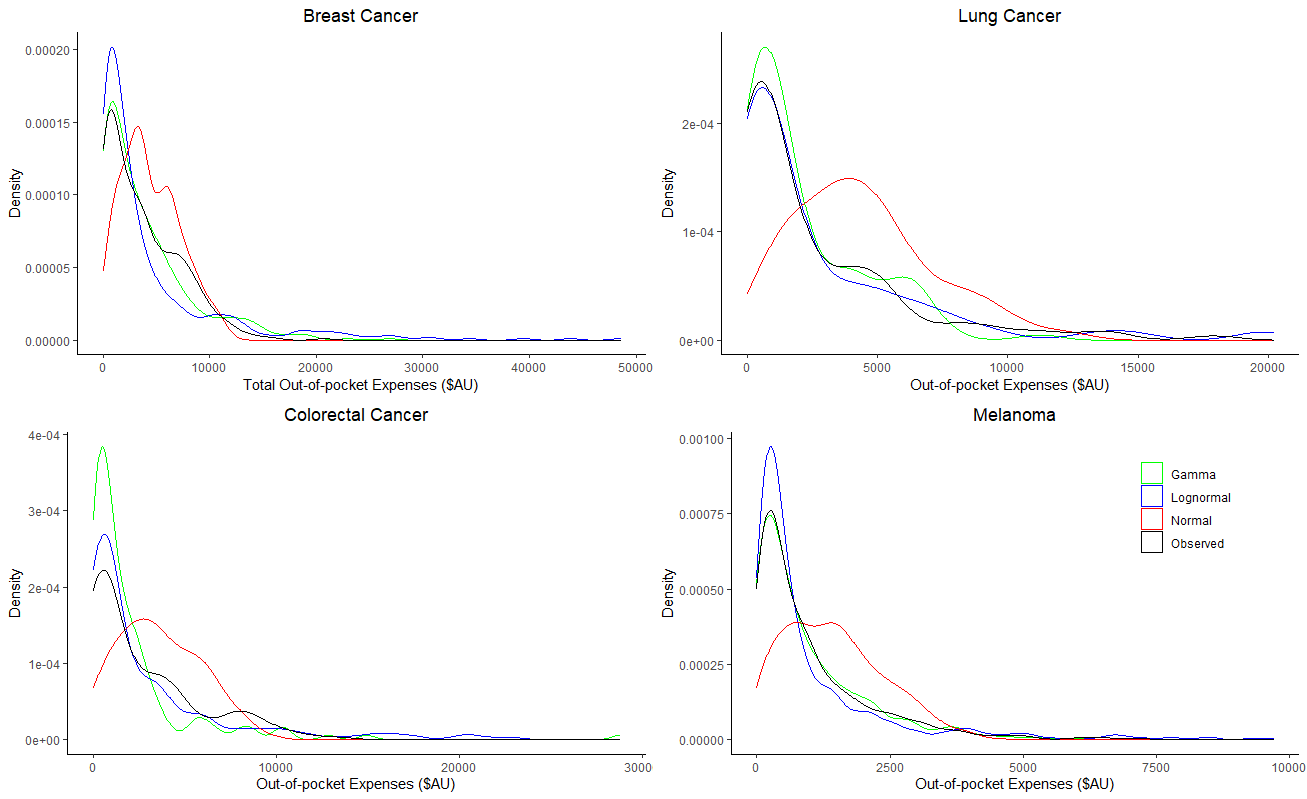


**Additional File 1. Density plots for the tested gamma, lognormal and normal distributions.** Observed values fitted better a gamma distribution. Diagnostic tests confirmed the appropriateness of the family and link parameters. Variables from a full model (year of diagnosis, age group, sex, marital status, level of education, private health insurance status (yes/no), body mass index, drinks per week and self-health assessment (poor, good and excellent)) were excluded based on their statistical significance and the Akaike Information Criterion (AIC). All analyses were performed using R. Statistical significance was considered at p<0.05.
