# Supplementary material for: Out-of-pocket medical expenses compared across five years for patients with one of five common cancers in Australia
Source: BMC Cancer. 2021 Sep 25;21:1055. doi: 10.1186/s12885-021-08756-x (PMC8466922; doi:10.1186/s12885-021-08756-x)
Supplement: Supplementary file 2 — Additional file 2. Calculation of the Fisher indices. [file 12885_2021_8756_MOESM2_ESM.docx]

**Additional File 2. Calculation of the Fisher indices.** The Fisher price index (PF) evaluates the year-to-year average change of patients’ out-of-pocket (OOP) cost (28,29). Fisher price indices were calculated using the formula below, where n is the total number of items, $p_{i}^{t}$ is the price for item *i* in year t, and $q_{i}^{t}$ its quantity, $P_{F}^{t}$ is the Fisher price index in year *t* :

$$P_{L}^{t}=\frac{\sum_{i=1}^{n} p_{i}^{t}q_{i}^{0}}{\sum_{i=1}^{n} p_{i}^{0}q_{i}^{0}}$$

$$P_{P}^{t}=\frac{\sum_{i=1}^{n} p_{i}^{t}q_{i}^{t}}{\sum_{i=1}^{n} p_{i}^{0}q_{i}^{t}}$$

$$P_{F}^{t}= \sqrt{P_{L}^{t}P_{P}^{t}}$$

The quantity index ( $Q_{F}^{t}$ ) summarizes quantities of Medicare items consumed after adjusting for changes in price. It is calculated as:

$$Q_{L}^{t}=\frac{\sum_{i=1}^{n} p_{i}^{0}q_{i}^{t}}{\sum_{i=1}^{n} p_{i}^{0}q_{i}^{0}}$$

$$Q_{P}^{t}=\frac{\sum_{i=1}^{n} p_{i}^{t}q_{i}^{t}}{\sum_{i=1}^{n} p_{i}^{t}q_{i}^{0}}$$

$$Q_{F}^{t}= \sqrt{Q_{L}^{t}Q_{P}^{t}}$$

|  |  | **2011-2015** | | **2011** | | **2012** | | **2013** | | **2014** | | **2015** | |
| --- | --- | --- | --- | --- | --- | --- | --- | --- | --- | --- | --- | --- | --- |
| **Cancer Type** |  | **Mean** | **SD** | **Mean** | **SD** | **Mean** | **SD** | **Mean** | **SD** | **Mean** | **SD** | **Mean** | **SD** |
| **All cancers** | *Observed* | 2406 | 3143 | 1911 | 2906 | 2482 | 3128 | 2585 | 3305 | 2551 | 3179 | 2518 | 3152 |
|  | *Predicted^+^* | 2489 | 1932 | 2290 | 1867 | 2453 | 1907 | 2469 | 1867 | 2558 | 1953 | 2715 | 2070 |
| **Breast** | *Observed* | 3651 | 3477 | 2480 | 2943 | 3443 | 3126 | 4113 | 3642 | 4160 | 3722 | 3869 | 3565 |
|  | *Predicted^+^* | 3653 | 2023 | 3305 | 1746 | 3560 | 2012 | 4000 | 1989 | 3692 | 2292 | 3605 | 1892 |
| **Colorectal** | *Observed* | 2548 | 3019 | 2060 | 2620 | 3288 | 3812 | 2323 | 2649 | 2533 | 3071 | 2710 | 2995 |
|  | *Predicted^+^* | 2725 | 2620 | 2081 | 1366 | 2832 | 2170 | 3222 | 3742 | 2820 | 2409 | 2467 | 2253 |
| **Lung** | *Observed* | 2422 | 3380 | 2253 | 1855 | 2261 | 2974 | 2104 | 3741 | 2362 | 2707 | 3103 | 4723 |
|  | *Predicted^+^* | 2442 | 1812 | 2224 | 1634 | 2136 | 1672 | 2706 | 1781 | 2624 | 2084 | 2325 | 1825 |
| **Melanoma** | *Observed* | 1001 | 1224 | 651 | 1012 | 1133 | 1407 | 1088 | 1227 | 1049 | 1166 | 1129 | 1280 |
|  | *Predicted^+^* | 1003 | 414 | 956 | 405 | 980 | 399 | 1044 | 424 | 1002 | 414 | 1043 | 430 |
| **Prostate** | *Observed* | 4126 | 4112 | 3444 | 3955 | 4329 | 4348 | 5106 | 4360 | 3596 | 3804 | 4347 | 3934 |
|  | *Predicted^+^* | 4269 | 1848 | 4179 | 1767 | 4395 | 1833 | 4332 | 1752 | 4147 | 2025 | 4297 | 1915 |
