# Supplementary material for: Out-of-pocket medical expenses compared across five years for patients with one of five common cancers in Australia
Source: BMC Cancer. 2021 Sep 25;21:1055. doi: 10.1186/s12885-021-08756-x (PMC8466922; doi:10.1186/s12885-021-08756-x)
Supplement: Supplementary file 3 — Additional file 3. Adjusted and unadjusted mean out-of-pocket expenses per year per cancer type. [file 12885_2021_8756_MOESM3_ESM.docx]

**Additional File 3. Adjusted and unadjusted mean out of pocket expenses per year per cancer type.** Out of pocket expenses significantly increased over the year 2011-2015 for patients with breast cancer and melanoma. ^+^ Adjusted like this: a) All cancers: health insurance, sex, age, level of education, cancer type and Year b) Breast and colorectal cancer: Health insurance, age, year c) Lung and Prostate cancer: Health insurance and year d) melanoma: health insurance, year, level of education.

| **Variable** | **Coeff** | **Ratio** | **Extra Cost (AU$)** | **Sign** |
| --- | --- | --- | --- | --- |
| **Health Insurance** |  |  |  |  |
| No (ref) |  |  | 803.38 (1494.82) |  |
| Yes | 1.32 | 2.76 | 2217 | *** |
| **Sex** |  |  |  |  |
| Female (ref) |  |  | 2441 (2967) |  |
| Male | 0.01 | 0.01 | 21 |  |
| **Age Category** |  |  |  |  |
| < 50 (ref) |  |  | 2452 (3335) |  |
| 50-60 | -0.05 | -0.05 | -121 |  |
| > 60 | -0.01 | -0.01 | -34 |  |
| **Level of Education** |  |  |  |  |
| High - (ref) |  |  | 2930 (3190.8) |  |
| Medium | -0.06 | -0.06 | -172 |  |
| Low | -0.25 | -0.22 | -657 | *** |
| **Cancer Type** |  |  |  |  |
| Breast (ref) |  |  | 3707 (3474) |  |
| Colorectal | -0.24 | -0.21 | -797 | * |
| Lung | -0.27 | -0.24 | -882 | * |
| Melanoma | -1.23 | -0.71 | -2627 | *** |
| Prostate | 0.22 | -0.24 | -899 | * |
| **Year** |  |  |  |  |
| 2011 (ref) |  |  | 1962 (2861) |  |
| 2012 | 0.52 | 0.68 | 1340 | *** |
| 2013 | 0.48 | 0.61 | 1200 | *** |
| 2014 | 0.49 | 0.63 | 1234 | *** |
| 2015 | 0.53 | 0.70 | 1379 | *** |
