# Supplementary material for: Out-of-pocket medical expenses compared across five years for patients with one of five common cancers in Australia
Source: BMC Cancer. 2021 Sep 25;21:1055. doi: 10.1186/s12885-021-08756-x (PMC8466922; doi:10.1186/s12885-021-08756-x)
Supplement: Supplementary file 4 — Additional file 4. Generalized Liner Model (GLM) results for all cancers. [file 12885_2021_8756_MOESM4_ESM.docx]

**Additional File 4. Generalized Liner Model (GLM) results for all cancers**. Coeff: Regression coefficient; Ratio:exp(Coeff)-1; Extra cost: Extra cost paid with respect to the mean of the reference group (ref) in each categorical variable; Sign: Statistical Significance; * < 0.05; ** < 0.001; *** < 0.0001

| **Breast cancer** | Cost ratio | Std. Error | z value | P value |
| --- | --- | --- | --- | --- |
| (Intercept) | 2.81 | 1.40 | 2.01 | 0.044 |
| Private HI | 18.68 | 3940.00 | 0.01 | 0.996 |
| Age 50-60 | 0.00 | 1.25 | 0.00 | 1.000 |
| Age >60 | 0.18 | 1.25 | 0.15 | 0.883 |
| Year 2012 | 0.47 | 1.45 | 0.32 | 0.749 |
| Year 2013 | 18.70 | 6230.00 | 0.00 | 0.998 |
| Year 2014 | 0.49 | 1.45 | 0.34 | 0.735 |
| Year 2015 | 18.68 | 7544.00 | 0.00 | 0.998 |
| Private HI: Year2012 | -18.24 | 3940.00 | -0.01 | 0.996 |
| Private HI: Year2013 | -18.68 | 8167.00 | 0.00 | 0.998 |
| Private HI: Year2014 | -17.82 | 3940.00 | -0.01 | 0.996 |
| Private HI: Year2015 | -18.64 | 9684.00 | 0.00 | 0.999 |
|  |  |  |  |  |
| **Colorectal** | Cost ratio | Std. Error | z value | P value |
| (Intercept) | 1.98 | 0.53 | 3.71 | 0.000 |
| Year 2012 | 1.35 | 1.15 | 1.18 | 0.240 |
| Year 2013 | 1.73 | 1.14 | 1.51 | 0.130 |
| Year 2014 | 1.45 | 1.15 | 1.27 | 0.205 |
| Year 2015 | 17.59 | 2292.76 | 0.01 | 0.994 |
|  |  |  |  |  |
| **Lung** | Cost ratio | Std. Error | z value | P value |
| (Intercept) | 2.64 | 1.04 | 2.55 | 0.011 |
| Year 2012 | 17.93 | 4179.09 | 0.00 | 0.997 |
| Year 2013 | -0.20 | 1.27 | -0.16 | 0.877 |
| Year 2014 | 0.58 | 1.45 | 0.40 | 0.690 |
| Year 2015 | 17.93 | 3780.13 | 0.01 | 0.996 |
|  |  |  |  |  |
| **Prostate** | Cost ratio | Std. Error | z value | P value |
| (Intercept) | 2.52 | 0.39 | 6.41 | 0.000 |
| Year 2012 | 0.86 | 0.71 | 1.22 | 0.224 |
| Year 2013 | 0.98 | 0.82 | 1.19 | 0.233 |
| Year 2014 | 1.64 | 1.08 | 1.52 | 0.130 |
| Year 2015 | 1.86 | 1.08 | 1.72 | 0.085 |
|  |  |  |  |  |
| **Melanoma** | Cost ratio | Std. Error | z value | P value |
| (Intercept) | 2.81 | 0.33 | 8.63 | 0.000 |
| Year 2012 | -0.15 | 0.44 | -0.35 | 0.730 |
| Year 2013 | 1.64 | 0.78 | 2.10 | 0.036 |
| Year 2014 | 0.52 | 0.56 | 0.93 | 0.350 |
| Year 2015 | 1.07 | 0.67 | 1.60 | 0.110 |
