# Supplementary material for: Out-of-pocket medical expenses compared across five years for patients with one of five common cancers in Australia
Source: BMC Cancer. 2021 Sep 25;21:1055. doi: 10.1186/s12885-021-08756-x (PMC8466922; doi:10.1186/s12885-021-08756-x)
Supplement: Supplementary file 5 — Additional file 5. First part of two-part model by cancer type. [file 12885_2021_8756_MOESM5_ESM.docx]

**Additional File 5. First part of two-part model by cancer type.** Ratio:exp(Coeff)-1; Std Error: Standard error

|  |  | **Price Index** | | | | | **Quantity Index** | | | | |
| --- | --- | --- | --- | --- | --- | --- | --- | --- | --- | --- | --- |
|  |  | **2011** | **2012** | **2013** | **2014** | **2015** | **2011** | **2012** | **2013** | **2014** | **2015** |
|  |  |  |  |  |  |  |  |  |  |  |  |
| **All cancers** | **Bulk Bill services %** | 0.47 | 0.49 | 0.49 | 0.51 | 0.48 |  |  |  |  |  |
|  | **all services** | 1 | 1 | 1.01 | 0.99 | 1.03 | 1 | 1.46 | 1.46 | 1.34 | 1.19 |
|  | **Attendances** | 1 | 1.04 | 1.07 | 1.05 | 1.12 | 1 | 1.46 | 1.39 | 1.37 | 1.03 |
|  | **Diagnostic and Therapeutic** | 1 | 1 | 0.99 | 0.98 | 1.01 | 1 | 1.44 | 1.45 | 1.33 | 1.23 |
|  | **Imaging** | 1 | 0.95 | 0.96 | 1.03 | 1.04 | 1 | 1.49 | 1.49 | 1.14 | 1.06 |
|  | **Pathology** | 1 | 1 | 1.04 | 0.99 | 1.04 | 1 | 1.56 | 1.54 | 1.5 | 1.29 |
|  | **PBS** | 1 | 1.09 | 1.14 | 1.1 | 1.12 | 1 | 1.37 | 1.34 | 1.44 | 1.04 |
| **Breast** | **Bulk Bill services %** | 0.43 | 0.46 | 0.46 | 0.46 | 0.49 |  |  |  |  |  |
|  | **all services** | 1 | 1.01 | 1.03 | 1.09 | 1.13 | 1 | 1.56 | 2.23 | 2.22 | 1.13 |
|  | **Attendances** | 1 | 1.09 | 1.13 | 1.08 | 1.11 | 1 | 1.49 | 1.93 | 2.05 | 0.98 |
|  | **Diagnostic and Therapeutic** | 1 | 1.01 | 1.01 | 1.11 | 1.16 | 1 | 1.53 | 2.30 | 2.33 | 1.18 |
|  | **Imaging** | 1 | 1.01 | 1.04 | 1.11 | 1.09 | 1 | 1.64 | 2.28 | 1.63 | 1.05 |
|  | **Pathology** | 1 | 0.90 | 0.97 | 0.95 | 1.00 | 1 | 1.75 | 2.19 | 2.35 | 1.14 |
|  | **PBS** | 1 | 1.20 | 1.39 | 1.12 | 1.28 | 1 | 1.38 | 1.42 | 1.72 | 0.80 |
| **Colorectal** | **Bulk Bill services %** | 0.34 | 0.48 | 0.48 | 0.53 | 0.46 |  |  |  |  |  |
|  | **all services** | 1 | 1.04 | 1.13 | 0.99 | 1.07 | 1 | 1.53 | 2.30 | 2.33 | 1.18 |
|  | **Attendances** | 1 | 0.99 | 1.07 | 1.01 | 1.10 | 1 | 1.60 | 1.27 | 1.17 | 0.68 |
|  | **Diagnostic and Therapeutic** | 1 | 1.04 | 1.15 | 0.99 | 1.05 | 1 | 1.31 | 1.50 | 1.29 | 1.04 |
|  | **Imaging** | 1 | 0.94 | 1.01 | 0.71 | 0.96 | 1 | 2.25 | 1.08 | 1.20 | 1.04 |
|  | **Pathology** | 1 | 1.15 | 1.21 | 1.02 | 1.09 | 1 | 1.60 | 1.50 | 1.30 | 1.04 |
|  | **PBS** | 1 | 0.92 | 0.97 | 1.15 | 1.06 | 1 | 1.57 | 2.54 | 2.21 | 1.16 |
| **Lung** | **Bulk Bill services %** | 0.43 | 0.49 | 0.46 | 0.49 | 0.38 |  |  |  |  |  |
|  | **all services** | 1 | 1.24 | 1.13 | 1.06 | 1.24 | 1 | 1.20 | 1.34 | 1.49 | 1.80 |
|  | **Attendances** | 1 | 1.13 | 1.27 | 1.07 | 1.12 | 1 | 1.83 | 1.58 | 1.64 | 2.19 |
|  | **Diagnostic and Therapeutic** | 1 | 1.28 | 0.97 | 0.98 | 1.24 | 1 | 0.93 | 1.13 | 1.46 | 1.58 |
|  | **Imaging** | 1 | 0.94 | 1.01 | 1.11 | 1.23 | 1 | 0.96 | 1.50 | 1.25 | 1.62 |
|  | **Pathology** | 1 | 1.41 | 1.40 | 1.33 | 1.46 | 1 | 1.17 | 1.40 | 1.42 | 1.95 |
|  | **PBS** | 1 | 0.88 | 1.02 | 1.24 | 1.39 | 1 | 1.47 | 1.20 | 1.10 | 1.00 |
| **Prostate** | **Bulk Bill services %** | 0.48 | 0.45 | 0.48 | 0.50 | 0.48 |  |  |  |  |  |
|  | **all services** | 1 | 0.99 | 1.01 | 0.96 | 0.94 | 1 | 1.42 | 1.12 | 0.91 | 1.22 |
|  | **Attendances** | 1 | 1.07 | 1.02 | 1.10 | 1.07 | 1 | 1.31 | 0.96 | 0.83 | 1.07 |
|  | **Diagnostic and Therapeutic** | 1 | 0.98 | 1.01 | 0.94 | 0.92 | 1 | 1.42 | 1.14 | 0.93 | 1.24 |
|  | **Imaging** | 1 | 0.93 | 0.85 | 0.95 | 0.92 | 1 | 1.56 | 1.13 | 0.78 | 1.09 |
|  | **Pathology** | 1 | 1.04 | 1.13 | 1.02 | 1.00 | 1 | 1.44 | 1.09 | 0.95 | 1.26 |
|  | **PBS** | 1 | 0.93 | 0.86 | 0.83 | 0.86 | 1 | 1.14 | 0.84 | 1.20 | 1.15 |
| **Melanoma** | **Bulk Bill services %** | 0.63 | 0.58 | 0.55 | 0.56 | 0.51 |  |  |  |  |  |
|  | **all services** | **1** | **1.02** | **1.06** | **1.07** | **1.12** | 1 | 1.61 | 1.34 | 1.19 | 1.15 |
|  | **Attendances** | 1 | 0.98 | 1.02 | 1.07 | 1.20 | 1 | 1.44 | 1.27 | 1.14 | 0.96 |
|  | **Diagnostic and Therapeutic** | 1 | 1.12 | 1.14 | 1.10 | 1.00 | 1 | 1.91 | 1.42 | 1.21 | 1.44 |
|  | **Imaging** | 1 | 0.82 | 0.98 | 0.92 | 1.07 | 1 | 1.24 | 1.03 | 0.93 | 0.70 |
|  | **Pathology** | 1 | 1.03 | 1.05 | 1.06 | 1.12 | 1 | 1.73 | 1.77 | 1.65 | 1.79 |
|  | **PBS** | 1 | 1.24 | 1.24 | 1.18 | 1.15 | 1 | 1.48 | 1.32 | 1.29 | 1.13 |
