# Supplementary material for: Out-of-pocket medical expenses compared across five years for patients with one of five common cancers in Australia
Source: BMC Cancer. 2021 Sep 25;21:1055. doi: 10.1186/s12885-021-08756-x (PMC8466922; doi:10.1186/s12885-021-08756-x)
Supplement: Supplementary file 6 — Additional file 6. Price and Quantity Index. [file 12885_2021_8756_MOESM6_ESM.docx]

**Additional File 6. Price and Quantity Index.** To evaluate whether prices were rising for each category of service, the year-to-year average change from the base year (2011) was calculated using the Fisher Price Index. Progressively increasing indexes over time indicate higher prices o larger number of services from 2011 to 2015.

|  |  | **2011** | | | **2012** | | | **2013** | | | **2014** | | | **2015** | | |
| --- | --- | --- | --- | --- | --- | --- | --- | --- | --- | --- | --- | --- | --- | --- | --- | --- |
|  |  | **Mean** | **Median** | **SD** | **Mean** | **Median** | **SD** | **Mean** | **Median** | **SD** | **Mean** | **Median** | **SD** | **Mean** | **Median** | **SD** |
| **Breast** | **Attendances** | 283.63 | 173.75 | 328.28 | 455.89 | 255.96 | 629.72 | 506.99 | 359.61 | 510.34 | 450.52 | 397.39 | 442.09 | 408.26 | 228.15 | 443.37 |
|  | **Dxn and Therapy** | 1527.47 | 865.58 | 2027.88 | 1796.12 | 1840.26 | 1759.39 | 2263.23 | 1556.92 | 2389.81 | 2739.68 | 2040.05 | 2851.34 | 2286.82 | 1493.87 | 2441.97 |
|  | **Dxn Imaging** | 211.79 | 89.58 | 301.45 | 277.65 | 109.06 | 410.44 | 300.43 | 182.81 | 369.05 | 230.65 | 107.28 | 354.21 | 274.09 | 86.94 | 405.69 |
|  | **Pathology** | 206.20 | 27.65 | 305.69 | 378.12 | 289.55 | 382.73 | 418.80 | 408.08 | 429.72 | 439.54 | 427.82 | 423.99 | 411.07 | 421.65 | 427.08 |
|  | **PBS** | 501.75 | 371.82 | 460.60 | 625.93 | 472.84 | 554.39 | 629.93 | 455.40 | 589.49 | 480.02 | 338.87 | 418.85 | 522.58 | 296.99 | 504.11 |
| **Colorectal** | **Attendances** | 270.45 | 115.43 | 394.28 | 486.52 | 360.11 | 553.20 | 280.83 | 114.07 | 373.75 | 324.20 | 103.63 | 490.08 | 305.99 | 80.81 | 484.63 |
|  | **Dxn and Therapy** | 1623.85 | 1370.53 | 1699.82 | 2142.13 | 1764.58 | 2352.75 | 1475.18 | 461.28 | 1799.63 | 1199.22 | 282.38 | 1767.87 | 1624.07 | 1387.38 | 1876.27 |
|  | **Dxn Imaging** | 182.69 | 0.00 | 322.23 | 188.93 | 0.00 | 410.44 | 97.92 | 0.00 | 150.13 | 120.74 | 0.00 | 210.13 | 126.10 | 0.00 | 267.21 |
|  | **Pathology** | 186.22 | 80.33 | 242.16 | 347.44 | 48.11 | 639.23 | 211.87 | 98.83 | 321.83 | 212.04 | 80.10 | 263.07 | 241.53 | 201.90 | 338.60 |
|  | **PBS** | 414.63 | 286.57 | 419.44 | 497.41 | 402.80 | 434.23 | 504.93 | 440.13 | 436.95 | 655.92 | 481.16 | 617.37 | 508.91 | 381.80 | 445.13 |
| **Lung** | **Attendances** | 303.46 | 325.71 | 348.64 | 504.41 | 120.25 | 770.68 | 408.40 | 0.00 | 850.18 | 473.27 | 154.09 | 827.44 | 486.01 | 112.86 | 926.80 |
|  | **Dxn and Therapy** | 1064.04 | 660.42 | 1118.68 | 978.34 | 144.74 | 1781.60 | 1136.53 | 0.36 | 2117.60 | 1058.12 | 44.80 | 1762.07 | 1252.08 | 24.44 | 2183.25 |
|  | **Dxn Imaging** | 198.27 | 79.92 | 317.98 | 200.67 | 23.12 | 327.79 | 226.14 | 0.00 | 336.38 | 213.55 | 10.99 | 383.00 | 316.88 | 54.38 | 585.31 |
|  | **Pathology** | 218.96 | 195.77 | 255.08 | 255.33 | 57.63 | 393.89 | 274.23 | 0.00 | 492.64 | 219.07 | 90.65 | 365.98 | 347.52 | 0.00 | 641.17 |
|  | **PBS** | 621.16 | 475.18 | 522.41 | 463.95 | 419.51 | 291.14 | 376.55 | 278.25 | 338.44 | 544.48 | 415.61 | 530.75 | 650.81 | 425.25 | 870.65 |
| **Melanoma** | **Attendances** | 163.94 | 97.81 | 238.52 | 230.62 | 158.65 | 287.75 | 215.46 | 139.35 | 274.73 | 234.09 | 180.50 | 270.26 | 229.86 | 141.19 | 253.65 |
|  | **Dxn and Therapy** | 313.09 | 79.60 | 670.19 | 562.95 | 138.31 | 978.54 | 434.37 | 97.08 | 799.37 | 348.98 | 52.72 | 659.13 | 507.88 | 102.60 | 913.42 |
|  | **Dxn Imaging** | 75.88 | 0.00 | 163.27 | 64.95 | 0.00 | 149.92 | 67.40 | 0.00 | 120.52 | 55.60 | 0.00 | 128.55 | 69.40 | 0.00 | 131.51 |
|  | **Pathology** | 38.38 | 0.00 | 91.37 | 50.86 | 0.00 | 93.86 | 49.07 | 0.00 | 90.50 | 50.72 | 0.00 | 119.04 | 66.76 | 0.00 | 147.49 |
|  | **PBS** | 253.56 | 187.82 | 287.76 | 339.36 | 258.93 | 358.78 | 387.52 | 236.38 | 429.25 | 391.12 | 315.33 | 330.46 | 365.04 | 272.85 | 362.80 |
| **Prostate** | **Attendances** | 208.28 | 174.39 | 195.02 | 285.11 | 250.81 | 238.34 | 296.09 | 267.45 | 240.76 | 243.96 | 166.04 | 240.57 | 303.75 | 203.39 | 337.53 |
|  | **Dxn and Therapy** | 3339.27 | 2185.13 | 3464.48 | 3654.73 | 1610.96 | 3692.22 | 4379.54 | 4656.38 | 3736.86 | 2823.70 | 1111.96 | 3280.43 | 3650.16 | 2796.31 | 3304.77 |
|  | **Dxn Imaging** | 198.99 | 69.46 | 295.42 | 188.75 | 83.91 | 246.29 | 176.10 | 142.29 | 200.00 | 156.88 | 67.27 | 207.77 | 178.87 | 147.48 | 188.10 |
|  | **Pathology** | 216.22 | 104.63 | 290.70 | 306.64 | 221.35 | 334.22 | 359.08 | 279.44 | 355.93 | 269.48 | 115.92 | 338.78 | 340.74 | 254.93 | 332.33 |
|  | **PBS** | 265.44 | 201.16 | 297.57 | 301.29 | 181.53 | 452.99 | 293.42 | 219.59 | 301.34 | 328.11 | 254.77 | 320.90 | 249.08 | 199.90 | 220.00 |
